# Seizing the moment: the potential of PrEP choice and innovation to transform HIV prevention

**DOI:** 10.1002/jia2.26498

**Published:** 2025-07-02

**Authors:** Heather‐Marie A. Schmidt, Mateo Prochazka, Heather Ingold, Sushena Reza‐Paul, Thato Chidarikire, Irvin Romyco, Michelle Rodolph

**Affiliations:** ^1^ Global HIV, Hepatitis and STIs Programme World Health Organization Geneva Switzerland; ^2^ UNAIDS Geneva Switzerland; ^3^ UNAIDS Regional Office for Asia and the Pacific Bangkok Thailand; ^4^ World Health Organization South Africa Office Pretoria South Africa; ^5^ World Health Organization Indonesia Office Jakarta Indonesia

**Keywords:** choice, differentiated service delivery, HIV prevention, long‐acting ARVs, PrEP, structural interventions

## Abstract

**Introduction:**

The potential of pre‐exposure prophylaxis (PrEP), as a highly effective and empowering HIV prevention intervention, has not yet been realized. Despite the recent acceleration in the scale‐up of oral PrEP, there is a substantial unmet PrEP need, and the world is not on track to meet the 2025 prevention targets. New PrEP products, and service delivery approaches, could support greater access, uptake, persistence and effective use. This commentary discusses how offering choice in PrEP products and service delivery innovations could transform global HIV prevention efforts.

**Discussion:**

Although oral PrEP accounts for almost all PrEP use to date, slow rollout and challenges in effective use and persistence have limited the global impact. Innovative products like long‐acting injectable cabotegravir and injectable lenacapavir can overcome some of the challenges associated with oral PrEP. Expanding PrEP choices is also essential for addressing diverse individual preferences and maximizing prevention outcomes. Real‐world evidence suggests that offering increased options can drive demand and increase coverage of prevention.

Equally critical is tailoring service delivery through differentiated service delivery (DSD) models that prioritize accessibility and user needs and preferences, including integration of PrEP within other valued services. DSD models, including peer‐led, pharmacy‐based and telehealth approaches, have demonstrated success and acceptability for oral PrEP, but innovation is needed to adapt to long‐acting injectable options. For example, regulatory and policy support are essential to support task‐sharing with community health worker involvement may enable broader reach.

Programmatic challenges, including PrEP product and service delivery costs, updating monitoring and evaluation and ensuring stakeholder support, must also be addressed. Scaling up new PrEP products using a precision prevention lens could help to optimize approaches for achieving impact.

**Conclusions:**

The new era of PrEP choice, with new long‐acting PrEP products and DSD options, presents countries with an extraordinary opportunity to amplify prevention access, achieve higher prevention coverage and drive the meaningful reductions in new HIV acquisitions needed to end the HIV epidemic. Without coordinated and concerted efforts within countries and supported at the global level to leverage choice and embed it within the HIV prevention response, we risk prolonging the HIV epidemic.

## 1 INTRODUCTION

Pre‐exposure prophylaxis (PrEP) is an effective, safe and empowering intervention offering individuals discretion and autonomy in HIV risk reduction and complementing other combination HIV prevention approaches [1, 2, 3, 4]. PrEP use is increasing annually with more than 6.2 million people estimated to have ever used PrEP [5] and 3.5 million people using PrEP in 2023 alone, an increase of 36% from the previous year [6]. However, with only 16% of the 21.2‐million‐person target for 2025 reached, scale‐up is slow, largely concentrated within a handful of countries and substantiates an enormous unmet need for PrEP [6]. Unless there is an unprecedented acceleration in both access and use, the potential for PrEP to turn around the HIV epidemic will not be realized.

For almost a decade, oral PrEP containing tenofovir disoproxil fumarate was the only PrEP option recommended by the World Health Organization (WHO) [7] and has accounted for almost all PrEP use worldwide. National HIV prevention programmes are beginning to provide access to new long‐acting PrEP options outside clinical trials, including the dapivirine vaginal ring (DVR) and long‐acting injectable cabotegravir (CAB‐LA), recommended by WHO in 2021 and 2022, respectively [7]. Additional PrEP products are rapidly moving along the pipeline, such as 6‐monthly injectable lenacapavir (LEN) and the dual prevention pill (DPP) [8]. Despite their own challenges, these newer PrEP products offer distinct advantages, including overcoming some of the challenges associated with oral PrEP. Evidence shows that choice‐based health interventions can significantly increase positive outcomes such as persistence and effective use [9].

Offering choice, in PrEP and other prevention products and the associated service delivery, could catalyse an HIV prevention revolution and be a key strategy to achieving the HIV prevention use targets. In this commentary, we discuss how leveraging new, alongside proven, PrEP products and service delivery options to offer choice may be the missing puzzle piece for maximizing uptake, effective use and persistence and harnessing the potential of PrEP on the HIV epidemic.

## 2 DISCUSSION

### 2.1 Choice in PrEP products

From the supermarket to the bedroom, not everyone likes or wants the same things so providing options that appeal to people who would benefit from PrEP is fundamental to facilitating choice.

For almost a decade, oral PrEP filled a critical gap in the HIV prevention market as a highly effective, safe and user‐controlled option for HIV prevention that is delivered in clinical and community settings, and by a range of providers [7]. In high‐income settings such as Australia, the United Kingdom and parts of the United States of America, even with high treatment and testing coverage, high PrEP uptake has been associated with population‐level decreases in new HIV acquisitions even despite declining condom use [10, 11, 12, 13, 14, 15, 16, 17, 18]. However, clients have experienced challenges using oral PrEP effectively and persistently complicating implementation and attenuating impact. Approximately 41% of people who start oral PrEP stop using it within the first 6 months, and these rates are higher in eastern and southern Africa at 47.5% [19].

The introduction of two long‐acting products, CAB‐LA, administered as an intramuscular gluteal injection every 2 months, and the DVR, an impregnated silicon ring worn in the vagina for 1 month, can overcome challenges of regular pill‐taking associated with oral PrEP, offering increased discretion and convenience, and few side effects [7].

Availability and access to the three WHO‐recommended PrEP products is patchwork. DVR rollout has been slow and primarily in eastern and southern African countries although Cambodia and Indonesia will begin implementation in 2025. CAB‐LA demonstrated very high efficacy and persistence was high within the trials [20, 21]. At the time of writing, programmatic CAB‐LA rollout has begun in low‐ and middle‐income countries (LMICs) including Zambia, Zimbabwe, Malawi, Ukraine and Eswatini.

Newer promising options are also moving along the antiretroviral (ARV)‐based prevention pipeline, including a 3‐monthly vaginal ring, the DPP and 1‐monthly oral pill [8]. The 6‐monthly injectable, LEN, will be the next PrEP product available. The PURPOSE 1 and PURPOSE 2 studies demonstrated a very high efficacy with HIV incidence reduced by 100% and 96%, respectively, compared with the background rate [22, 23]. These results have been met with enthusiasm from communities, governments and donors. LEN has enormous potential to make PrEP use and delivery simpler and more effective by overcoming some of the challenges associated with other PrEP options by offering high protection, infrequent 6‐monthly visits, no regular pill taking, and enhanced privacy and discretion. The challenges associated with LEN, including risks of drug resistance, and implementation considerations remain to be evaluated.

As each PrEP product has a unique set of attributes, offering advantages and disadvantages depending on the person and context, and as preferences may change with time or circumstance, there is no perfect product. The best PrEP product is one that an individual wants to use and will use well. Studies across regions and populations have demonstrated the diversity of stated preferences for different PrEP products, with a substantial interest in long‐acting injectable PrEP products that could help meet unmet need [24−26]. In a survey in 11 LMICs in Asia, 23.3% and 26.8% of gay men and other men who have sex with men and transgender women participants, respectively, chose a long‐acting injectable as their preferred PrEP product, with the other options being oral PrEP (daily or event‐driven dosing) (44/5% and 47.8%), a 1 month pill (23.3% and 16.1%) and a removable implant (8% and 5.8%) [26]. In Brazil, injectable and implantable PrEP products were preferred [25]. This suggests that offering additional PrEP options could help meet this unmet need. Community organizations are campaigning to prioritize scaling up a spectrum of options to offer real choice to individuals, such as the African Women Prevention Community Accountability Board's HIV prevention choice manifesto [27]. Early implementation of CAB‐LA also suggests a diversity of preferences. In Zimbabwe, over 72% of users on oral PrEP are reported to have switched to CAB‐LA [28], 30% of CAB‐LA users within the first 2 months of implementation in Zambia had transitioned from oral PrEP and 70% were PrEP naïve [29], and 56% of participants in the SEARCH study in Uganda and Kenya chose CAB‐LA compared with 53% choosing oral PrEP [30].

There is compelling evidence for different populations and regions that increasing prevention and PrEP options, increases overall prevention coverage, that is the proportion of people using HIV prevention options. In Australia, the scale‐up of PrEP as an additional HIV prevention option among gay men and other men who have sex with men was associated with an increase in overall prevention coverage (70% in 2017 to 75% in 2021), despite declines in condom use [12]. In Kenya and Uganda, the SEARCH study demonstrated that the option to choose and switch between CAB‐LA, oral PrEP and post‐exposure prophylaxis (PEP) increased biomedical prevention coverage by five times compared with the standard of care (oral PrEP and PEP only) [30]. In South Africa, early data from implementation science and pilot projects showed that offering choice improved demand for PrEP, with sites offering three PrEP products (oral PrEP, the DVR and CAB‐LA) having notably higher PrEP uptake than those offering one or two products [31]. Taken together, these findings provide compelling, real‐world evidence that not only can new products that are aligned to the needs and preferences of individuals attract new clients, but that choice can increase the use of prevention overall, including existing options. The downstream benefits of increased prevention use would be reduced new HIV acquisitions. Introducing CAB‐LA in eastern and southern African countries is predicted to reduce HIV incidence by 29% over a 20‐year time horizon and this benefit is partly attributable to the use of PrEP increasing from 28% to 46% by having an additional effective PrEP option [32].

### 2.2 Choice in PrEP service delivery

Person‐centred access and service delivery models are crucial to facilitate PrEP use in a multi‐product environment. Differentiated service delivery (DSD) models for PrEP are person and community‐centred, allowing for increased availability, accessibility, acceptability and affordability when implemented at scale, and may support greater uptake, effective use and persistence [33]. An important, and too often underutilized DSD approach, is PrEP integration within services needed and valued by people who may benefit from PrEP, including pre‐ and post‐natal services.

Over the last 5 years, accelerated by the COVID‐19 pandemic, DSD models have played an increasing role in supporting oral PrEP access and uptake with a wide range of models implemented including pharmacy‐based, peer‐led, telehealth and self‐care models [33, 34]. Studies have shown both the importance of DSD models in increasing service access and acceptability and the diversity of preferences, which shows it is unlikely that any single service delivery model can meet the needs of all individuals [26, 35, 36, 37, 38]. Discrete choice experiments in the PrEP APPEAL study indicated clear preferences for community‐based services and longer follow‐up intervals among transgender women and gay and other men who have sex with men in Asia‐Pacific, though many individuals were satisfied with facility‐based models [26]. Similarly, in South Africa, PrEP initiation at a pharmacy or mobile clinic was valued equally to initiating PrEP at a clinic [38]. In Thailand, a country that pioneered peer‐led services, 82% of PrEP users in 2021 received their PrEP through key population‐led services [39]. For female sex workers in sub‐Saharan Africa, there is a need for more services with flexible clinic hours, multi‐month dispensing, community‐based options, involving peers in service delivery, and other DSD features to maximize uptake, effective use and persistence [37].

The introduction of long‐acting injectable PrEP options such as CAB‐LA and LEN may present challenges for implementation in existing DSD models. As an intramuscular injectable, CAB‐LA requires trained and authorized providers and a private space that may not easily be provided through pharmacy‐based, telehealth or peer‐led services [40]. The recent recommendation from the WHO on HIV self‐testing (HIVST) for starting, continuing and/or restarting oral PrEP and the DVR is enabling for community‐based and self‐care models but does not include CAB‐LA, given a paucity of evidence at the time of the recommendation to support the use of HIVST with long‐acting injectables [41]. Early programmatic implementation of CAB‐LA has only been delivered in clinical settings. LEN, though administered as a subcutaneous injection every 6 months, may have similar regulatory hurdles to delivery through DSD models. With policy and regulatory support, accompanied by training and task sharing, diverse delivery of long‐acting injectables is possible and can be strengthened by co‐delivery with other services in the community, including long‐acting contraceptives. Community health workers have been successfully tasked with administering injectable medications from COVID‐19 vaccinations to contraceptives [42] and there is proof of concept for peer‐led administration of CAB‐LA in Washington, DC [43].

### 2.3 What does offering choice mean for PrEP programming?

With the increasing availability of effective PrEP options and services, countries will need to balance the advantages of offering individual choice with potential programmatic challenges including cost, procurement, and identifying and delivering a mix of options. This means that PrEP choice is likely to look different in different contexts.

In the short term, rapid scale‐up of PrEP will have important budget implications and countries will need to balance the introduction of new PrEP options with available resources. Modelling has suggested that if CAB‐LA is scaled up to increase overall PrEP use and is delivered at a similar cost as oral PrEP, it is likely to be cost‐effective [32]. However, newer PrEP options, particularly prior to generic availability, are substantially more expensive than the lowest drug‐only oral PrEP ($3.09 per 30 pills or $37 USD per year for daily use) [44] with the not‐for‐profit price for 2025 for CAB‐LA for low income, least developed and eastern and southern African countries at £20.70/vial (or $160 USD per year) [45]. Many countries do not benefit from this lowest price, particularly middle‐income countries outside Africa, and for countries, it may be unaffordable for domestic procurement [46]. However, if PrEP can be successfully scaled up to close to the UNAIDS targets, the costs could be reduced substantially. The cost of goods analysis predicts that with economies of scale, the injectable component of generic LEN could be available for as little as $96 USD per person per year for 1 million persons and $41 USD per person per year if procured for 10 million people [47] and there could be substantially lower service delivery costs with only two visits a year.

With higher product costs, PrEP may be justifiably targeted to, or rely on self‐selection of, populations and/or geographies with a higher HIV incidence for cost‐effectiveness [48, 49]. But countries should not discount DSD models, including community‐led, for cost‐effectiveness and epidemiological impact [50].

To end the HIV epidemic, countries and donors should look beyond the cost‐effectiveness of a single product and consider the possible impacts of offering choice (increased access, coverage and reduction in new HIV acquisitions). With the expanding array of PrEP products, countries will need to look at the optimal product mix to prioritize for scale‐up. The concept of “precision prevention,” that is using the available epidemiological and programme evidence to deliver the right mix of prevention options to the right people, at the right locations, at the right time and at the right cost, provides a programmatic framework to prioritize the available prevention interventions [51].

Enabling choice requires government support and leadership at all levels, funding for product choice and delivering options, and ensuring that users are informed and empowered to make choices about PrEP use and engage with health services. With the rollout of multiple PrEP products, there is an urgent need to train providers, and develop client‐facing materials, to present the available PrEP options, their advantages and disadvantages, in a way that someone can select the option that best meets their needs and preferences [7]. Studies are testing the feasibility of delivering choice, with early positive data for feasibility in public health facilities [52].

Accelerating PrEP scale‐up and the adoption of new PrEP products and DSD models will require proactive and pragmatic policy support and ownership by government and stakeholders, including communities. Task sharing and community‐based models of PrEP service delivery, including telehealth and pharmacies, may require regulatory or policy changes at the national level to allow for different cadres of workers to deliver different PrEP products across various settings.

Little is currently known about the real‐world patterns of use and switching with multiple PrEP products available, limiting the evidence for guidance on choice. Improved country completion of the global AIDS monitoring indicators could provide this evidence. Monitoring and evaluation systems will need to be updated to track and interpret emerging patterns of PrEP use, stopping and switching between products, to contribute towards the global evidence and facilitate programme service delivery, procurement and quality assurance as new products are added in countries.

Offering choice and addressing inequities in access to effective interventions is central to a human‐rights‐based approach for HIV prevention. True choice cannot exist without a supportive environment, including enabling policies and laws, a lack of stigma and discrimination, as well as community awareness and PrEP literacy. Lessons can be learned from the family planning field, which has a longer history of providing choice according to women's needs and preferences and in measuring switching [53].

## 3 CONCLUSIONS

We are embarking on a new era of PrEP choice. The increasing availability of diverse PrEP options, including long‐acting products like CAB‐LA and LEN, that are aligned to the needs and preferences of users and supported by innovative DSD models, can transform the HIV response. Coordinated efforts among stakeholders at national and global levels are needed to centre HIV prevention and PrEP choice in programme planning and financing, ensure structural and individual factors limiting PrEP programmes are addressed and create enabling environments that empower individuals to make informed choices. By leveraging choice, countries can amplify prevention access, achieve higher prevention coverage and drive reductions in new HIV acquisitions. Our challenge is to realize this potential. If we miss this chance, it may not come again, and the consequences for the HIV epidemic beyond 2030 could be disastrous.

## COMPETING INTERESTS

The authors declare no competing interests.

## AUTHORS’ CONTRIBUTIONS

H‐MAS drafted the initial manuscript. All authors contributed to the review and finalization of the manuscript.

## DISCLAIMER

The views expressed in this manuscript are the authors’ own and do not necessarily reflect the views of the World Health Organization (WHO) or UNAIDS.

## Data Availability

Data sharing is not applicable to this article as no datasets were generated or analysed during the current study.
